# Changes in household hygiene behaviors and multiple childhood health outcomes following health-behavioral education and health-care facility support: a pre–post intervention study

**DOI:** 10.3389/fpubh.2026.1862959

**Published:** 2026-07-08

**Authors:** Mansour Abdu Al-Taj, Ashwaq Abdulrahman Al-Taj

**Affiliations:** 1 Department of Community Medicine, Faculty of Medicine and Health Sciences, Sana’a University, Sana’a, Yemen; 2 Migration and Refugee Studies Center, Sana’a University, Sana’a, Yemen

**Keywords:** acute diarrhea, acute malnutrition, behaviors, children, dietary diversity, internally displaced persons, intervention, vaccination

## Abstract

**Background:**

In crisis and displacement settings, disruptions in safe water, sanitation, health service access, and caregiver knowledge often increase children’s risk of acute watery diarrhea, malnutrition, and missed preventive care. This study assessed the impact of six months of integrated health-promotion activities at the household level and support for health facilities on changes in household behaviors and child health indicators in crisis contexts.

**Methods:**

A pre- and post-intervention study was conducted among 895 households of internally displaced persons (IDPs) and host communities in four Yemeni governorates. The intervention included health-promotion activities at the household level and support for health facilities in the catchment areas of IDPs, including medical supplies, incentives for health workers, and outreach activities for remote areas. The effect size of the intervention was estimated using Generalized Estimating Equations (GEE).

**Results:**

Significant improvements were observed in household chlorine use for drinking water treatment (adjusted prevalence ratio (APR) = 1.69; 95% CI: 1.25–2.28), cooking area cleanliness (APR = 1.09; 95% CI: 1.01–1.18), and the proportion of toilets free from visible fecal matter (APR = 1.10; 95% CI: 1.02–1.19). In addition, reductions in acute watery diarrhea (APR = 0.76; 95% CI: 0.60–0.97) and improvements in dietary diversity (APR = 1.83; 95% CI: 1.30–2.58) and vaccination coverage (APR = 1.16; 95% CI: 1.07–1.26) were observed among children. However, no significant improvements were found in the proper disposal of child feces or in the prevalence of acute malnutrition among children.

**Conclusion:**

The improvement in certain behavioral factors and child health indicators emphasizes the effectiveness of the integrated community health promotion and health facilities-based interventions. However, the increase in the prevalence of acute malnutrition following the intervention than before highlights the need for continued efforts, combining food security or livelihood initiatives with such projects to address vulnerabilities, particularly among IDPs and host communities.

## Introduction

Access to safe water, adequate sanitation, and proper hygiene (WASH) remains a major global public health challenge. Globally, 3.4 billion people still lacked safely managed sanitation services; 1.7 billion did not have a designated handwashing facility with soap and water at home (1), and at least 1.7 billion people used drinking-water sources contaminated with feces (2). Limited access to clean water and sanitation facilities increases exposure to infectious diseases and adversely affects nutritional status, particularly among vulnerable populations in low-resource settings (3–6). According to the World Health Organization, unsafe WASH conditions are responsible for an estimated 1.4 million preventable deaths annually, mainly due to diarrhea, undernutrition, and acute respiratory infections (7).

Conflict, violence, and persecution have displaced millions of people worldwide (8), many of whom reside in overcrowded camps or temporary settlements with inadequate access to essential services. Internally displaced persons (IDPs) are particularly vulnerable due to damage to WASH infrastructure (9, 10). Moreover, access to healthcare services is frequently limited in these settings (11, 12), restricting the availability of essential medical care and routine immunization programs (13). Food insecurity represents an additional challenge, as displacement disrupts food supply systems, livelihoods, and income-generating opportunities, thereby limiting access to adequate and nutritious food (14, 15). Consequently, displaced children experience an elevated risk of morbidity and mortality (16, 17).

Several studies conducted in low- and middle-income countries have demonstrated that hygiene promotion interventions can improve household hygiene behaviors (18) and reduce the incidence of childhood diarrhea (19), and malnutrition (20). Similarly, outreach community health programs have been associated with increased utilization of health services and improved child health outcomes (21). However, the effectiveness of such interventions may vary depending on the context, implementation approach, and existing health system capacity. In fragile and conflict-affected settings, where environmental and socioeconomic challenges are particularly severe, evidence on the impact of integrated hygiene and healthcare support interventions remains limited (22).

Yemen is one of the countries suffering from prolonged conflict, resulting in the displacement of nearly 4.8 million people (23). Limited access to WASH services remains a major public health concern in Yemen, where United Nations Children’s Fund estimates that more than 16 million people, including 8.47 million children, are in urgent need of WASH assistance (24). Additionally, food insecurity and poverty, which constrain children’s consumption of a balanced and nutritious diet, have been identified as significant determinants of poor health outcomes among children in the country (25, 26). Recent evidence suggests that children living in IDP settlements and host communities experience a substantial burden of acute diarrhea and acute malnutrition (27, 28). Despite extensive humanitarian investments in WASH and health programs (29), there is a scarcity of studies evaluating the effectiveness of integrated interventions on household hygiene practices and multiple childhood health outcomes. Therefore, this study aimed to assess changes in household hygiene behaviors and childhood health outcomes following the implementation of a health-behavioral education program combined with healthcare facility support in vulnerable communities in Yemen (IDPs and host community). The findings are expected to contribute to the evidence base on integrated community and health system interventions in humanitarian settings and inform future public health programming aimed at improving child health among vulnerable populations in Yemen and similar contexts.

## Methods

### Study design, setting, population

An uncontrolled before-and-after intervention study was conducted between February and December 2024. Repeated cross-sectional surveys were carried out one month prior to the intervention (baseline) and two months following its completion (endline) to assess changes in selected household hygiene behaviors and child health indicators between the pre-intervention and post-intervention periods. The study focused on IDP households and host communities living in Taiz, Hadramout, Marib, and Al-Jawf governorates in Yemen. Inclusion criteria were limited to households with a mother and at least one child aged between 6 months and 50 months, families willing to participate in the study, and those who decided not to leave the study area during the following 12 months.

### Sample size and sampling technique

The sample size was determined based on the number of households required for the baseline survey (27). A formula for a single proportion was used for this calculation, considering a prevalence of acute malnutrition of 16%, as reported in the Yemeni National Health and Demographic Survey 2013, along with a 95% confidence interval and a significance level of 0.05. The initial sample size was estimated at 206 households and adjusted for the cluster sampling design using a design effect of 2 (30), resulting in a required sample size of 412 households. To ensure adequate representation of both IDPs and host communities and to account for an anticipated 5% nonresponse rate, the target sample size was increased to 900 households. The final sample was allocated proportionally between the two population groups, with 627 households selected from IDP communities and 268 households from host communities.

For both the baseline and endline surveys, a two-stage cluster sampling approach was used. First, 30 clusters were selected from a list of 27 previously enumerated areas prepared by the Human Access Team through systematic random sampling. After, approximately 30 households within each selected cluster were chosen using the World Health Organization Expanded Programme on Immunization (EPI) sampling method (31).

### Data collection

Data collection for the baseline survey was conducted by 30 trained female data collectors. To minimize potential bias, the female data collectors from the baseline survey were replaced by a new group of 30 trained female data collectors for the endline survey. Information regarding mothers, households, child diarrhea, dietary intake, and vaccinations was self-reported by mothers during face-to-face interviews using a structured questionnaire. Observations of household drinking water treatment practices, cooking area cleanliness, food covering, and latrine hygiene were recorded using structured observation checklists. Children’s Mid-Upper Arm Circumference (MUAC) was measured using UNICEF measuring tapes with the assistance of their mothers. MUAC was used to assess acute malnutrition because it is a simple, rapid, low-cost, and reliable anthropometric measure that can be easily applied in community and humanitarian settings (32). MUAC measurement involved bending the child’s left arm and placing the measuring tape from the tip of the shoulder to the tip of the elbow, then marking the midpoint. The measurement was taken to the nearest 0.1 centimeter by placing the tape across the mid-upper arm without tightening or loosening it.

## Intervention

The present study is informed by the Theory of Planned Behavior (TPB), a widely used framework for understanding and promoting health-related behaviors (33). According to TPB, behavior is mainly determined by an individual’s intention to perform that behavior, which is influenced by attitudes toward the behavior, subjective norms, and perceived behavioral control. Interventions that target these determinants can increase the likelihood of adopting and sustaining positive behaviors. In the context of WASH, nutrition, and child health, behavior change communication activities may improve caregivers’ attitudes toward recommended practices, strengthen supportive social norms, and enhance their confidence in implementing these behaviors.

The interventions integrated health education awareness, and health care services. The health education awareness sessions conducted from March to September 2024 aimed to disseminate five critical messages: handwashing at the five key moments, maintaining a safe water chain from collection to storage and treatment (boiling and chlorination), ensuring kitchen and food hygiene, promoting latrine cleanliness and safe disposal of feces, and encouraging a balanced diet. These sessions were facilitated by 50 trained male and female community volunteers who utilized illustrated posters to enhance understanding. During the six-month intervention, approximately 6,000 household visits and 2,000 public sessions were carried out. Each participating family received two hygiene kits, which included powdered soap, body soap, two jerry cans, towels, and chlorine tablets, thus providing essential resources to support the adoption of healthier practices within the community. The health component included support for seven health facilities by providing essential drugs, incentives for health workers, and basic medical equipment in the catchment area of the IDPs. This support aimed to offer health services free of charge to the IDPs and affected host communities. Additionally, two mobile clinics, staffed by three health workers each, were assigned to deliver health services to IDPs in Al-Jawf and certain areas in Marib where no health facilities were available.

### Study outcomes

The primary outcomes of the study were changes in selected household hygiene behaviors. These included the proportion of households that treated drinking water through boiling or chlorination, maintained clean food preparation areas, covered remaining food, kept toilet free of visible fecal matter, reported no unpleasant odors in toilet, had no flies observed around toilet, and safely disposed of child feces in toilet or through other appropriate methods.

The secondary outcomes were changes in child health and nutrition indicators, including the prevalence of acute diarrhea and acute malnutrition, vaccination coverage, and minimum dietary diversity.

Acute watery diarrhea was defined as the passage of three or more loose, watery stools in 24 h during the two weeks preceding the interview (34). Children whose MUAC was less than 12.5 cm were considered acutely malnourished (35). For children aged 6 to 23 months, minimum dietary diversity was defined as consuming foods and beverages from at least five out of eight specified food groups during the previous day. These groups include: (1) breast milk; (2) grains, roots, tubers, and plantains; (3) pulses (beans, peas, lentils), nuts, and seeds; (4) dairy products (milk, infant formula, yogurt, cheese); (5) flesh foods (meat, fish, poultry, organ meats); (6) eggs; (7) Vitamin A-rich fruits and vegetables; and (8) other fruits and vegetables. For children older than 23 months, minimum dietary diversity was defined as receiving food from at least four out of seven food groups (36). Children who received all the recommended vaccines by the Ministry of Health and the World Health Organization according to their age were considered fully vaccinated.

The demographic and household characteristics of the participants include the mother’s age group classified into five groups (≤ 25 years or from 26 to 30 years or 31 to 39 years or ≥ 40 years), the mother’s educational level as no education, primary, secondary or higher, and the mother’s work as a housewife or employee. The age of the child was classified into two groups: 6 to 23 months and 24 months and above. The sex of the child was categorized as male or female. The type of house was categorized as an independent house/flat or tent, the source of drinking water was considered improved if it was from a government or private network or from a protected well or treated water and unimproved if it was from tankers or an open, unprotected well, and the sanitation was improved if toilet was connected to septic tanks or a public sewer network and not improved if it was an open pit or a shared toilet or no toilet.

### Statistical analysis

Data cleaning and analysis were performed using Stata 16. Proportions were employed to characterize categorical variables. Respondent and household characteristics at baseline and endline were compared using the chi-square test. Associations between the intervention period and study outcomes were assessed using GEE with a Poisson distribution, log link, robust standard errors, and an exchangeable correlation structure to account for clustering at the community level. Crude and adjusted prevalence ratios (PR) with 95% confidence intervals (CI) were estimated. Adjusted analyses controlled for maternal age, maternal education, household type (IDP or host community), and current place of residence, family size, and access to improved sanitation facilities.

## Results

This analysis included 895 households at baseline and 894 at endline. Significant differences were observed between the two surveys in maternal age (*p* = <0.001), maternal education (*p* = <0.001), family size (*p* = 0.010), and access to improved sanitation (*p* = 0.029). No significant differences were observed in maternal employment, child age and sex, type of housing, access to improved drinking water, displacement status (Table 1).

**Table 1:** Socio-demographic, household, and child characteristics of the study population at baseline and endline surveys.

Characteristics	Baseline (*n* = 895)		Endline (*n* = 894)		*p* value
	Number	Percent	Number	Percent	
Mother’s age (years)					
≤ 25	226	25.2	138	15.4	<0.001
26–30	260	29.1	238	26.6	
31–39	250	27.9	273	30.5	
≥ 40	159	17.8	245	27.4	
Mother’s education					
No education	418	46.7	396	44.3	<0.001
Primary	272	30.4	221	24.7	
Secondary and above	205	22.9	277	31.0	
Mother’s work					
No	823	92.0	834	93.3	0.281
Yes	72	8.0	60	6.7	
Child’s age (month)					
6–23	575	64.3	559	62.5	0.451
≥ 24	320	35.7	335	37.5	
Child’s sex					
Male	457	51.1	456	51.0	0.982
Female	438	48.9	438	49.0	
Type of house					
Independent/Flat	431	48.2	467	52.2	0.084
Hut/Tent	464	51.8	427	47.8	
Family size					
3	77	8.6	118	13.2	0.010
4	117	13.1	116	13.0	
5	142	15.9	152	17.0	
≥ 6	559	62.4	508	56.8	
Access to improved drinking water					
Yes	404	45.1	402	45.0	0.941
No	491	54.9	492	55.0	
Access to improved sanitation					
Yes	637	71.2	677	75.7	0.029
No	258	28.8	217	24.3	
Type of family					
Internally displaced	627	70.1	627	70.1	
Host	268	29.9	267	29.9	
Current place of residence (governorate)					
Taiz	240	26.8	239	26.7	
Hadramout	148	16.5	148	16.5	
Marib	357	39.9	357	39.9	
Al-Gawf	150	16.8	150	16.8	

Following the implementation of the interventions, significant improvements were observed in four key household hygiene practices. Households were 69% more likely to treat drinking water with chlorine at endline compared with baseline (APR = 1.69; 95% CI: 1.25–2.28). Additionally, the prevalence of maintaining a clean cooking area increased by 9% (APR = 1.09; 95% CI: 1.01–1.18), while the prevalence of covering leftover food increased by 6% (APR = 1.06; 95% CI: 1.01–1.11). The prevalence of toilets free from visible fecal matter also increased by 10% (APR = 1.10; 95% CI: 1.02–1.19). No statistically significant changes were observed in the remaining household hygiene practices assessed (Table 2).

**Table 2:** Changes in household water treatment, food hygiene and sanitation practices from pre- to post-intervention.

Household behaviors	Pre %	Post %	CPR	*p* value	APR	*p* value
Drinking water treated by boiling	59.3	62.0	1.04 (0.86–1.23)	0.607	1.02 (0.87–1.19)	0.778
Drinking water treated with chlorine	29.1	50.0	1.72 (1.26–2.34)	0.001	1.69 (1.25–2.28)	0.001
Clean cooking area	69.4	77.3	1.11 (1.02–1.22)	0.015	1.09 (1.01–1.18)	0.030
Clean cooking utensils	79.4	85.6	1.08 (1.01–1.15)	0.024	1.05 (0.99–1.12)	0.087
Remaining food properly covered	83.6	89.8	1.07 (1.02–1.13)	0.003	1.06 (1.01–1.11)	0.013
Toilet free from visible feces	67.3	75.0	1.12 (1.05–1.21)	0.001	1.10 (1.02–1.19)	0.012
Toilet free from foul odor	63.7	69.0	1.10 (0.99–1.23)	0.073	1.07 (0.97–1.19)	0.171
Toilet free from flies	62.5	66.2	1.08 (0.97–1.21)	0.153	1.06 (0.94–1.18)	0.340
Proper disposal of child feces	30.3	35.1	1.16 (0.87–1.54)	0.306	1.14 (0.86–1.49)	0.350

%, percent; CPR, crude prevalence ratio; APR, adjusted prevalence ratio.

Regarding child health outcomes, the prevalence of acute watery diarrhea was significantly lower at endline than at baseline, with children being 24% less likely to experience diarrhea following the intervention (APR = 0.76; 95% CI: 0.60–0.97). Although the prevalence of acute malnutrition increased between baseline and endline, this change was not statistically significant (APR = 1.22; 95% CI: 0.83–1.78). Significant improvements were observed in child vaccination coverage and dietary diversity. The prevalence of children who had received all age-appropriate vaccinations increased by 16% between baseline and endline (APR = 1.16; 95% CI: 1.07–1.26). Similarly, the prevalence of achieving minimum dietary diversity was 1.83 times higher at endline than at baseline (APR = 1.83; 95% CI: 1.30–2.58) (Table 3).

**Table 3:** Changes in the prevalence of acute watery diarrhea, acute malnutrition, vaccination coverage, and food diversity among children from pre- to post-intervention.

Variables	Pre %	Post %	CPR	*p* value	APR	*p* value
Acute watery diarrhea	36.4	27.2	0.74 (0.59–0.94)	0.014	0.76 (0.60–0.97)	0.027
Acute malnutrition	16.7	19.6	1.16 (0.78–1.71)	0.461	1.22 (0.83–1.78)	0.308
Received all age-appropriate vaccinations	72.2	85.3	1.18 (1.09–1.27)	<0.001	1.16 (1.07–1.26)	<0.001
Achieved Minimum Dietary Diversity	20.8	38.7	1.87 (1.32–2.64)	<0.001	1.83 (1.30–2.58)	0.001

%, percent; CPR, crude prevalence ratio; APR, adjusted prevalence ratio.

## Discussion

The integrated intervention was associated with improvements in several household hygiene, child health, and nutrition indicators. Among the household hygiene, the largest improvement was observed in the treatment of drinking water with chlorine. This finding is likely attributable to both the distribution of water treatment materials and the health education messages promoting safe water handling and treatment practices. Interestingly, no significant improvement was observed in the treatment of drinking water by boiling. The lack of improvement in water boiling practices in this study may be attributed to insufficient financial resources to cover the cost of fuel. Similar interventions have reported differing findings, with some studies indicating improvements in household water treatment practices (37), while others found no significant association (38). Our findings highlight that combining the provision of WASH supplies with behavior change communication is more effective in improving household water treatment practices than education alone, particularly in humanitarian settings where access to safe water is limited.

The observed improvement in cooking area cleanliness, and the proper covering of remaining food may reflect increased attention to food preparation environments following exposure to the intervention, leading households to adopt practices that reduce the accumulation of dirt and other potential sources of food contamination. In contrast, no significant improvements were observed in the cleanliness of cooking utensils. The present findings are consistent with previous food hygiene intervention studies conducted in Bangladesh (39, 40), which reported heterogeneous effects across targeted behaviors. However, findings from a randomized controlled trial in Nepal demonstrated significant improvements across all targeted food hygiene indicators (41). This discrepancy may be attributable to a ceiling effect in the present study, where the high baseline prevalence of these practices limited the potential for further improvement compared with the Nepalese study.

Among the sanitation-related indicators assessed, the intervention resulted in a significant improvement in the proportion of toilets that were free from visible feces, while no significant changes were observed for toilets being free from foul odor, free from flies, or for the proper disposal of child feces. The improvement in toilet cleanliness may indicate that the intervention successfully influenced behaviors related to routine toilet maintenance and cleaning practices. The absence of similar improvements in other sanitation indicators may indicate that these outcomes are influenced by factors beyond household cleaning behavior alone. For example, foul odor and the presence of flies are often associated with structural and environmental conditions such as inadequate ventilation, poor pit design, high temperatures, and ineffective waste containment. The lack of improvement in the safe disposal of child feces in this study may be attributed to the absence of toilet in some households and a shortage of water necessary for maintaining hygiene after using toilet or disposing of feces properly. This study is consistent with studies that have reported positive impacts of similar intervention on toilet cleanliness (37, 42). Furthermore, evidence from a recent systematic review indicated that behavior change interventions may contribute to improvements in latrine quality, although no corresponding effect was observed for the safe disposal of child feces (43).

The reduction in acute watery diarrhea observed at endline may be attributable to improvements in household WASH practices promoted through the intervention. Increased use of chlorine for household water treatment, improved food hygiene practices, and better sanitation conditions likely reduced children’s exposure to enteric pathogens and interrupted fecal–oral transmission pathways. This finding is consistent with evidence indicating that targeted interventions can reduce acute diarrhea among children under five years of age (44–47). However, these findings contrast with those of previous studies that reported limited or no significant reduction in diarrheal incidence following similar interventions (38, 48, 49). Unexpectedly, the prevalence of acute malnutrition increased following the intervention period. This finding should be interpreted with caution, as acute malnutrition is influenced by multiple factors beyond WASH interventions, including food security, infectious diseases, and socioeconomic conditions. Remarkably, the study design accounted for the seasonality of cholera by conducting both the pre- and post-intervention assessments during the winter season, when suspected cholera cases are typically at their lowest levels. Nevertheless, the cholera outbreak that occurred between May and September 2024 may have contributed to the observed increase in acute malnutrition among children. Several reports have indicated that diarrhea can contribute to the development of acute malnutrition in children (50–52).

Furthermore, the inclusion of support to health facilities in remote IDP settlements, and the deployment of mobile clinics may have improved age-appropriate vaccination coverage by reducing geographical and logistical barriers to immunization services. The intervention was also associated with an improvement in minimum dietary diversity among children. This improvement may reflect increased caregiver awareness of appropriate child feeding practices, likely supported by nutrition-related messaging delivered through the intervention. Evidence from nutrition interventions indicates that behavior change communication and caregiver education can improve complementary feeding practices, including dietary diversity among young children (53).

The study has several limitations that should be acknowledged. Firstly, the post-intervention survey was conducted in a relatively short period following the completion of the interventions, which may not allow for a thorough assessment of the long-term impacts of the intervention. Additionally, the absence of a control group that was not exposed to the intervention limits the ability to draw definitive conclusions about the effectiveness of the interventions. Without this comparison, it is challenging to determine whether observed changes in indicators were directly attributable to the intervention or influenced by other external factors. To address these limitations, a cluster randomized trial design is recommended to assess the long-term impact of these integrated interventions.

## Conclusion

The study highlights several important contributions regarding the health education program. It effectively improved household hygiene practices, leading to a distinguished reduction in acute diarrhea cases and improved food diversity among children. However, the intervention did not significantly reduce acute malnutrition, suggesting that child undernutrition is influenced by factors beyond WASH and behavior change alone, such as household food insecurity and socioeconomic conditions. These findings highlight the need for integrated approaches. Policymakers should therefore combine WASH interventions with nutrition programs that improve household food access and strengthen services for vulnerable children. Furthermore, the importance of supporting health facilities and introducing mobile clinics have significantly increased vaccination coverage and may improve other indicators of access to and utilization of health services by vulnerable populations. However, this study did not assess the effectiveness of these supports in enhancing access to health services. Therefore, it is recommended that further research be conducted to evaluate the impact of supporting health facilities and mobile clinics on overall health access.

## Data Availability

The raw data supporting the conclusions of this article will be made available by the authors, without undue reservation.
